# How participation in Covid‐19 mutual aid groups affects subjective well‐being and how political identity moderates these effects

**DOI:** 10.1111/asap.12275

**Published:** 2021-10-21

**Authors:** Guanlan Mao, John Drury, Maria Fernandes‐Jesus, Evangelos Ntontis

**Affiliations:** ^1^ University of Sussex, Brighton, United Kingdom; ^2^ York St John University, York, United Kingdom; ^3^ Canterbury Christ Church University, Canterbury, United Kingdom; ^4^ The Open University, Milton Keynes, United Kingom UK

**Keywords:** Activism, covid‐19, groups, mental health, mutual aid, social cure, social identity, subjective wellbeing

## Abstract

Mutual aid groups have flourished during the Covid‐19 pandemic. However, a major challenge is sustaining such groups, which tend to decline following the initial upsurge immediately after emergencies. The present study investigates one possible motivation for continued participation: the well‐being benefits associated with psychological membership of groups, as suggested by the “social cure” approach. Interviews were conducted with 11 volunteers in a mutual aid group organized by ACORN, a community union and anti‐poverty campaigning organization. Through qualitative analysis, we show that participation provided well‐being in different ways: positive emotional experiences, increased engagement in life, improved social relationships, and greater sense of control. Participants also reported some negative emotional experiences. While all interviewees experienced benefits from participation, those who viewed their participation through a political lens were able to experience additional benefits such as feelings of empowerment. Moreover, the benefits conferred by a shared political identity appeared to be qualitatively different from the benefits conferred by other forms of shared identity. The interview data is used to hypothesize an overall process by which participants may come to attain a political identity via mutual aid. These findings have implications for how such groups retain their members and how authorities support these groups.

## INTRODUCTION

Self‐isolation, or quarantine, has been a key behavioral intervention to mitigate the spread of the Covid‐19 virus. In most countries, people testing positive for the virus, those displaying symptoms, and those who have had contact with others who tested positive have had to self‐isolate at home for periods of between 10 and 14 days at a time. The practical problems of self‐isolation—loss of income and accessing supplies—are among the most important reasons why most people required to self‐isolate do not do so consistently or for the required duration (Rubin et al., 2020; Smith et al., 2020a, 2020b; SPI‐B, 2020a; Webster et al., 2020).

Covid “mutual aid” and other community support groups have been critical in enabling many people to self‐isolate. The proliferation of such groups has been one of the most positive stories of the pandemic (Monbiot, 2020). Seebohm et al. (2013) define mutual aid as “self‐organizing groups where people come together to address a shared health or social issue through mutual support” (p. 391). Mutual aid is distinguished from charity by its ethic of mutualism and is typified by the slogan “solidarity, not charity,” erasing the traditional “hierarchical” divide between helper and helped in favor of “horizontalist” principles of democratic participation and reciprocity (Spade, 2019). Moreover, mutual aid often targets the underlying social causes behind hardship, and can also therefore constitute a form of political participation (Big Door Brigade, 2020).

During the Covid‐19 pandemic, which began in early 2020, the support provided by mutual aid groups has included shopping, collecting prescriptions, providing meals, dog‐walking, informational and emotional support, and involvement in running foodbanks (Solnit, 2020). Their flexibility and local connections meant they were better suited to play this role than centralized or official bodies (Tiratelli & Kaye, 2020). As of May 2021, there were at least 4317 mutual aid groups operating in the United Kingdom (Covid‐19 Mutual Aid UK, 2021).

A key question that arises from the phenomenon of Covid mutual aid and similar groups is how they can be sustained. Most countries have experienced a second wave of the virus, and at the time of writing the programs of mass vaccination are not completed. This means that there will be a continued need for nonpharmaceutical interventions, with an effective system of Find, Test, Trace, Isolate & Support being crucial (Independent SAGE, 2020). While upsurges in supportive behavior from members of the public are common after disasters (Drury, 2018; Drury et al., 2019; Fritz & Williams, 1957), such support typically declines over time (Kaniasty & Norris, 1999). There is both a practical and theoretical need to better understand the factors that can help such groups retain participants and organize themselves in a sustainable way.

One factor that might be important in continuing to motivate participants is the well‐being they get from volunteering as part of a group (Bowe et al., 2020; Gray & Stevenson, 2020). In the study described in this paper, we examine the question of whether participation in Covid mutual aid groups provides mental health and well‐being benefits to participants, and the processes by which it does so, through an interview study with 11 participants. We also examine whether the particular (political) group identity of volunteers adds value, and develop a novel hypothesis of how this might operate.

### Social support and mutual aid in disasters

The rapid rise of mutual aid groups in the Covid‐19 pandemic counters a pervasive stereotype surrounding disasters: that of social breakdown, whereby individuals revert to primitive self‐interested behavior (Auf der Heide, 2004). One of the first researchers to critique this misconception was Fritz (1961/1996; Fritz & Williams, 1957), who studied a wide range of disasters and found that panic was a rare response. Instead, disaster victims quickly organized and cooperated to help one another. The emergence of solidarity and the presence of social support have been observed in numerous disasters (Clarke, 2002; Drury, 2018; Grimm et al., 2014; Solnit, 2009).

Like other kinds of emergencies, the Covid‐19 pandemic saw a general rise in neighborliness (Addley, 2020; Monbiot, 2020) and high levels of reported and expected support (Office for National Statistics, 2020). As in the case of other types of emergency and disaster, a new sense of social unity was strongly associated with this rise in supportive behaviors (Parveen & McIntyre, 2020), with some studies finding a predictive association between the two (Stevenson et al., 2021; Vignoles et al., 2021).

The mutual support found after disasters typically declines over time (Kaniasty & Norris, 1999). These “altruistic communities” (Barton, 1969) typically run out of energy or resources, and are often undermined by state intervention (Kaniasty & Norris, 1999). The question of sustaining such groups has practical as well as theoretical importance. If we understand the psychological underpinnings of group membership, we can design interventions that help such groups sustain themselves. Recent work has examined some of the factors that enable such groups to sustain themselves (Ntontis et al., 2020; Tekin & Drury, 2021), including commemorations, group meetings, and alliances with other groups. Yet such work is preliminary, and has so far been addressed only to floods and fires, where, unlike Covid, the threat and the “victims” are both geographically and temporally bound. Here, we consider a further factor that could help sustain motivation for participation in the specific context of mutual aid groups, by drawing upon what is known about the well‐being benefits of psychological group membership.

### A social cure for the stresses of giving support in an emergency

In his survey of the effects of disasters, Fritz observed that many of those affected were counterintuitively mentally healthy, sometimes even healthier than before: for example, suicide rates consistently declined during disasters (Fritz, 1967/1996, pp. 43–44). To explain this, he hypothesized that disasters created a new set of social relations with several therapeutic properties: this “social therapy” effectively mitigated disaster's potentially traumatic features. By creating a “community of sufferers” (p. 28) characterized by solidarity, mutual support, and intimate relationships, a disaster had the potential to realize latent desires for personal and social transformation. Solnit (2009) documents a similar pattern of thriving community spirit and joy across five case studies. Solnit argues that these cases demonstrate human desires for “connection, participation, altruism, and purposefulness” (p. 338), which may be unmet in everyday life but are fulfilled within the disaster scenario.

The “social cure” approach in social psychology (Haslam et al., 2018; Jetten et al., 2017; Wakefield et al., 2019) suggests possible mechanisms for some of these observed benefits of the disaster community. The approach applies insights from social identity theory (Tajfel & Turner, 1979) and self‐categorization theory (Turner et al., 1987) to health and well‐being, with the core claim that social identities function as a psychological resource, contributing to recovery and resilience (Jetten et al., 2014). The number of social identities possessed by an individual, and the strength of those identifications, has been found to predict well‐being across a range of groups (Cruwys et al., 2013; Haslam et al., 2010, 2008). Specifically, shared social identities are hypothesized to contribute to well‐being by providing connection, meaning, support, and agency (Haslam et al., 2018). As such, activities that foster social identification may lead to greater well‐being. This has been found across a variety of contexts, but particularly relevant for the present study is a survey of community volunteers which found that volunteering predicted well‐being via increased community identification and social support (Bowe et al., 2020; see also Gray & Stevenson, 2020).

However, because a core contention of the social cure approach is that group identities *qua* group identities contribute to well‐being (Iyer et al., 2009), the question of whether different *types* of group identity can generate qualitatively different well‐being outcomes, or operate through different mechanisms, has yet to be fully examined. Some research suggests that participation in collective action groups can have benefits for well‐being (e.g., Cherniss, 1972; Klar & Kasser, 2009). However, as Vestergren et al. (2016) note, the underlying psychological processes remain underexamined in most of these studies: suggestions have included emotional expression (Foster, 2014), working toward something bigger than the self (Dwyer et al., 2019), and social support (Vestergren et al., 2019). A further suggestion is that action which brings about social change in line with the definition of an otherwise subordinated identity can produce well‐being, in the form of empowerment (Drury et al., 2015). The present study therefore investigates whether activism contributes to well‐being solely via “basic” social identity processes as posited by the social cure literature, or whether additional distinctive processes are at play related to the content of identity as “political” (i.e., achieving, or seeking to achieve, social change).

In this context, mutual aid is interesting to study because of its duality. Kavada (2020) notes that many groups who define themselves as “mutual aid” may have formed simply to help the vulnerable in a spirit of charity, rather than as conscious political projects. These ideological differences are reflected in the clashes both within and between groups over issues such as working with the police (Grayson, 2020) or local authorities (Dhillon, 2020). Therefore, the meaning of participation in mutual aid depends on the lens through which it is viewed: as (collective or individual) volunteering, or as political participation.

### The present study

Mental health in the United Kingdom deteriorated by 8.1% on average in the first two months of “lockdown,” taking into account prepandemic trajectories (Xu & Banks, 2020). The UN (2020) has stated that Covid‐19 contains the “seeds of a major mental health crisis” (p. 2). Faced with this, Fritz's “social therapy” may prove more important than ever. To sum up, the present study addresses three primary questions. First, what were the consequences of participation in mutual aid groups for the mental health and well‐being of participants? Second, via what processes did participation in mutual aid groups lead to these consequences? Third, did experiences of mutual aid differ based on participants’ political perspectives on the nature of the group? We leave open the possibility of developing new hypotheses, if suggested by the data, regarding further effect(s) of mutual aid participation on participants’ social identities and well‐being, besides the expected social cure processes.

We carried out 11 interviews with participants in the ACORN Covid‐19 mutual aid group. ACORN is a community organization, which defends the interests of renters and low‐income people experiencing housing issues via collective action (Cant, 2018). During the pandemic, ACORN shifted from their usual work to set up mutual aid networks in nine of their branches as part of their “Coronavirus Community Support” initiative (ACORN, 2020). Leaflets were distributed inviting members of the public to volunteer and/or request assistance. Each network consisted of a central spreadsheet moderated by administrative volunteers. These volunteers received requests for assistance via mobile hotline and entered these onto the spreadsheet; requests were then picked up by any available volunteers. Services included food and prescription deliveries, post collection, and social calls. Additionally, ACORN's volunteer pool provided a workforce for other organizations, such as food banks. By the 7th of April, over 1000 households across the country had received assistance from ACORN groups (ACORN UK, 2020a).

ACORN's initiative was selected for this study for several reasons. First, as a nationwide organization, it offered a large and diverse participant pool. Second, its shared spreadsheet system captured which volunteers were doing what, and so allowed for purposive recruitment of active volunteers. Third, ACORN are an overtly political organization, with a membership which predated the crisis. Therefore, existing members might be expected to (i) view their participation through a political lens and (ii) possess a preexisting shared identity as an ACORN member. At the same time, it was able to recruit a large number of new volunteers through its leafleting campaign. The presence of participants with a wide range of political perspectives allows us to better investigate the potential impact of such differing perspectives on participation.

## METHOD

### Participants

The criteria for inclusion in the study was participation in ACORN's Coronavirus Community Support initiative. We sought to recruit a sample that was relatively homogeneous and small, to allow us to examine the shared experiences of a particular group in depth (Braun & Clarke, 2013; Patton, 2002; Smith & Eatough, 2012). While sample sizes in qualitative studies are sometimes determined using the concept of “saturation,” for the present study this was not practicable: the interviews were conducted as the first UK lockdown was easing, a development that had the potential to cause the mutual aid campaign to die down or even end. It was therefore unfeasible to carry out, transcribe and analyze interviews with a view to carrying out further ones if necessary. We therefore determined a target sample size of 12 prior to sample collection, following Guest et al.'s (2006) suggestion that after 12 interviews, new themes are infrequent and code definitions are also fairly stable. This is particularly the case if interviews are structured/semistructured, and the sample is relatively homogeneous. Participants were recruited via several methods: personal contacts of the first author, advertising via social media groups, and approaching individuals who had been active on ACORN's organizing spreadsheet. As thanks, participants could vote for one of three charities to receive a share of a £25 donation (see Supporting Information for a list of the charities).

Fifteen individuals responded to our initial recruitment methods: of these, four dropped off after further contact and eleven were interviewed. Participants had engaged in various activities: managing the support line; organizing volunteers; delivering shopping, money, and prescriptions; and volunteering for allied organizations. Additionally, one participant (P2) had been involved in setting up their own local mutual aid network. All participants but one described joining the Community Support initiative from when it began in early April, soon after the first UK lockdown was announced on March 23; the remaining participant (P5) joined at the beginning of June. Levels of participation ranged from 2 h total to 25 h a week. Demographic details are outlined in Table 1.

**TABLE 1:** Participant demographics

Gender	Male	4
	Female	7
Age	Range	23–69
	Mean	37.27
Ethnicity	White	10
	East Asian	1
Level of education	A‐Level	3
	Undergrad	2
	Postgrad	6
ACORN branch	Manchester	2
	Brighton	9
ACORN membership	Prior ACORN member	4
	Joined for coronavirus community support campaign	7

### Interview procedure

Due to restrictions on in‐person research activities in place because of the pandemic, interviews were conducted remotely using the video‐conferencing software Zoom: video and audio were recorded and transcribed verbatim. Interviews were conducted between the 10th of May and the 6th of June, over a month after UK government lockdown regulations first came into effect, and during which time several easements to lockdown were introduced (Dunn et al., 2020). Interviews ranged from 30 min to an hour, with the mean average length being 40 min.

Interviews followed a semistructured format, guided by an interview schedule consisting of open questions supplemented with prompts and closed questions. A pilot interview was conducted to check the clarity and focus of the items, and minor refinements were made. The schedule was structured according to the following themes: (1) participants’ mental health and well‐being before participation in mutual aid, including any impact Covid‐19 had had on their well‐being (e.g., “Before you participated in ACORN's community support, how were you feeling in terms of your mental health?”); (2) the nature of the mutual aid activities they undertook (e.g., “What sorts of activities have you personally been involved in as part of ACORN's community support activities?”); (3) how participants felt immediately during those activities, and over a longer period of time (e.g., “Generally, since participating in community support have you noticed any impact, either positive or negative, to your general mood day‐to‐day?”); (4) any further changes in beliefs or behavior resulting from participation (e.g., “Has participating in community support led to any change in your beliefs about how society should be organized after Coronavirus?”). (See Supporting Information for full schedule.) Two months after the interview, participants were asked two yes–no follow‐up questions: whether at the time they viewed their participation as political, and whether they viewed it as such in retrospect.

### Ethical issues

Ethical approval was granted by the University of Sussex Sciences & Technology Cross‐Schools Research Ethics Committee on March 29, 2020 (Reference ER/GM408/2).

We confirm that we have reported all interview questions, data exclusions, and how we determined our sample size (Nosek et al, 2017).

### Analytic procedure

Data was analyzed using thematic analysis as outlined by Braun and Clarke (2006) and Joffe (2011). The method balanced “top‐down” and “bottom‐up” approaches, similar to Drury and Reicher (2005): the analyst approached the data with certain theoretical questions in mind (in this case, the above research questions), while remaining open to new issues arising, and aimed to avoid imposing their preconceptions by capturing the experiences of the interviewees as faithfully as possible. The first author read the transcript of each interview, highlighting and making notes for any statement seen as relevant to the above questions. No data from interviews was excluded. Extracts were grouped together and preliminary codes assigned to each group. These codes were placed into five superordinate themes: “Positive emotional experiences,” “Negative emotional experiences” “Increased sense of engagement in life,” “New or strengthened social relationships,” and “Greater sense of control.” Codes within each superordinate theme were constantly compared and merged to create themes (e.g., “Demonstrating effectiveness” was merged with “Political agency” to create “Empowerment”), with some codes being discarded. The entire dataset was then revisited to ascertain whether it was accurately captured by the coding scheme, with some subthemes being merged and others moved to a different superordinate theme (e.g., “Making a difference” was moved from “Increased sense of engagement in life” to “Positive emotional experiences”). Finally, a negative case analysis was conducted to identify themes that added another dimension or perspective to existing themes. Through this, a final theme was generated under “Negative emotional experiences,” “Witnessing difficult situations.” A further theme that did not fit into any existing superordinate themes, “Perceived Inadequacy of Government Response,” was also generated. Superordinate and subordinate themes are presented in Table 2.

**TABLE 2:** Superordinate and subthemes by participant

		P1	P2	P3	P4	P5	P6	P7	P8	P9	P10	P11
Existing ACORN member		Y	Y	N	Y	N	N	N	N	N	N	Y
Viewed participation in mutual aid as political		Y	Y	Y	Y	N	N	N	Y	N	N	Y
Level of involvement (hours per week)		4	28	1	1.5	0.5	2	1	10	25	14	25
**Superordinate themes**	**Subthemes**											
Positive emotional experiences	Receiving thanks	x	x	x		x	x	x		x		x
	Positive self‐concept				x	x	x	x				
	Making a difference	x	x	x	x	x		x	x	x	x	x
Negative emotional experiences	Stress			x		x		x				x
	Witnessing difficult situations	x		x	x			x	x	x	x	x
	Exploiting generosity									x	x	x
Increased sense of engagement in life	Structuring life	x			x	x	x	x	x		x	
	Sense of purpose	x		x				x	x	x	x	x
	Building the union	x										x
New or strengthened social relationships	New social bonds		x		x	x	x		x	x		x
	Camaraderie with volunteers	x							x	x		x
	Social support	x										
	Sense of community		x	x	x				x	x	x	
Greater sense of control	Agency	x	x	x	x				x	x		
	Empowerment	x	x		x							x
Perceived inadequacy of govt.		x	x	x	x		X	x	x		x	x

*Note*: A box marked “x” means that the participant described the relevant subtheme at least once.

The internal consistency of the analytic scheme was assessed through interrater reliability. An independent judge was then trained to apply the scheme: the rationale for each category was explained along with example extracts. The judge was given a sample of material to practice with until they stated they were comfortable doing so. They were then presented with 10% of all coded extracts and tasked with categorizing them, while blind to the analyst's coding decisions. From this a Cohen's Kappa of .87 was calculated, indicating “almost perfect” agreement (Landis & Koch, 1977).

As a form of validity check, an initial draft of the analysis was sent to five participants who featured prominently in the analysis (P1, P3, P4, P9, P11), with their prior agreement, in order to confirm that all interpretations of their extracts were fair and accurate (Dodson & Schmalzbauer, 2005). All of them chose not to suggest revisions to the interpretation or to add new comments.

### Analysis

The analysis is presented in two parts. The first part addresses the question of the consequences of participation in mutual aid groups for the mental health and well‐being of participants, and the processes involved in any such consequences. We present five superordinate themes, which capture the effects of participation in mutual aid on well‐being: generating *positive emotional experiences* as well as *negative emotional experiences*; *increased sense* of *engagement in life*; *new or strengthened social relationships*; and *greater sense of control* (see Table 2). For each area, the factors and processes leading to either a positive or negative impact on well‐being are explored as themes.

The second part of the analysis addresses whether the processes associated with well‐being differ based on participants’ political perspectives on the nature of the group. Here we argue experiences of participation were qualitatively different depending on how participants framed their activities. We hypothesize two connected processes by which participants may acquire a politicized identity. Throughout, extracts are presented which best represented the identified themes.

### Part 1: Effects of participation in Covid‐19 mutual aid groups

#### Positive emotional experiences

Participants reported experiencing a range of positive and negative emotions during their activities. All participants found at least some aspect of their participation to be immediately enjoyable. One participant explained this enjoyment in terms of doing things for others:

1. P: Erm, and I genuinely en‐, I sort, I'm the sort of person that genuinely enjoys doing something for somebody and recognise the mutual synchronicity, synchronicity that that gives, it's not just about helping somebody it is definitely, always gives you a buzz. (P7, M, 55, Brighton)

The participant quoted in extract 1 saw participation in the mutual aid group as enjoyable in and of itself. The reasons participants gave for this enjoyment fell into three major categories: first, their actions supported a view of themselves as virtuous moral agents; second, they contributed in a tangible way toward a meaningful goal; third, a sense of positive recognition from others. One participant expressed the first process straightforwardly:

2. I: Uh, has it had any impact on your self‐esteem, do you think?
 P: I think a little bit, you know? A little bit to ‐ it sort of makes you feel good about yourself, that you're doing something, in fact maybe it's got quite an outsized, sort of, impact on that, cause you can sort of think, you know? Yeah. I'm still a good person. (P4, F, 27, Brighton)

Here, to “feel good” is to “feel good about yourself”: that is to say, the validation of a *positive self‐concept*. Given the prevalent societal norm that helping others is good, such actions reinforce the view that one is a “good person.” One participant explicitly linked their enjoyment to their normative beliefs:

3. I: And how did you kind of generally feel during your participation in those activities? Did you feel sort of tired, did you feel that you were doing it begrudgingly or did you feel good about it? Did you feel positive?
 P: No, I love it. I love it. Absolutely. I really, I really enjoy ‐ I just, I've got a view that we should just help each other out a bit more. I think there's a lot of anger and hatred in the world because of this or that, you know, and, and I just think if we could just reach out to each other a little bit more. (P6, F, 59, Brighton)

By participating in the mutual aid group, the participant was able to view themselves as acting concordantly with their normative beliefs. Moreover, they were able to instantiate their beliefs concretely, and so bring the world closer to their beliefs about how it should be, a form of action which feels emotionally positive (Drury et al., 2005). This hints at the second process by which participants “felt good”: the idea that their actions had contributed toward something meaningful. The phrase *“making a difference”* was deployed by three separate participants:

4. I: And how did you feel during your participation in the, sort of, the campaign?
 P: Yeah, I felt, I felt, very, I thought it was very, like, I guess, it felt very rewarding, it felt like I was definitely trying, definitely, you know, participating in something that was making a difference to people who without ACORN or similar mutual aid groups would be in a very tough situation, um, and yeah, it made me feel very happy, and it was a very good use of my time. (P1, M, 23, Brighton)

In this extract, we can identify two components to the concept of “making a difference.” First, a clear and tangible outcome: without mutual aid groups, those being helped would be in a much worse situation. Second, an outcome that is perceived as worthwhile or valuable. While not all participants used this exact phrasing, overall 10 of them alluded to this concept. Participants were active in constructing their own sense of what was worthwhile, such as one individual who cleaned for a local food bank:

5. P: Something that I've really enjoyed about volunteering is that it's very, um, uh tangible…a thing as straightforward as scrubbing an oven, you, like, get it done, and then you're feeling, like, yeah, I've really helped because nothing can happen if, if, the [foodbank] isn't clean. Uh, so yeah, I do feel like I have made an impact. I guess I'm not on the frontline, so I'm not delivering parcels to people. So I don't get that kind of instant gratification I guess of people, uh, you know, being very grateful for the help or anything. Um, but I know that, I know that that is happening. So I'm quite happy to be kind of more behind the scenes. (P8, F, 28, Brighton)

In this case the participant was not “on the front line,” but was nonetheless able to locate their work as a vital part of a meaningful project.

Extract 5 also points to another prominent source of enjoyment which was cited by eight participants: the immediate gratification they attained through their interactions with those being helped. This was particularly the case when *receiving positive feedback*:

6. P: Oh, yeah, I've had lovely texts from people saying, you know, ‘you've really made such a difference. You know, now that you fixed my anxiety, I've been really worried. I've not been able to sleep knowing that I [inaudible] getting my food and my prescription’ and, yeah just little texts like that, and knowing that you really made a difference has been amazing. (P9, F, 38, Brighton)

Being thanked in this way creates a feeling of recognition: of being acknowledged for one's virtuous actions. Additionally, the feedback in this case revealed not only the material, but psychological impact of the participant's actions, and therefore increased their sense of having “made a difference.”

#### Negative emotional experiences

Participation was not universally enjoyable. In three cases, the feeling of making a difference was undercut by a perception that some were *exploiting others’ generosity*. One participant articulated their frustration during such encounters:

7. P: at times that was just like, yeah, it just, it just felt really special to be, to be able to help those people. Um, but there were other moments where it was frustrating. And I would talk to people who I would have good reason to think were, um, exploiting the generosity of the system that was in place? (P11, M, 24, Manchester)

The level of concentration and effort required of participants also led four participants to feel *stressed* during their participation:

8. P: You know, just the idea of having to put the mask on and arranging the payment and being really careful about wiping down the things you're touching. I mean, all of that is quite stressy. So, you know, it's not always been a stress reliever. Sometimes it's been, it's, sometimes it's added to this stress. (P7, M, 55, Brighton)

The fact that recipients of the mutual aid were often from a particularly vulnerable group (i.e., people who were elderly or shielding) raised the stakes: any mistake could have life‐threatening consequences. This in turn demanded a level of diligence which was stressful to uphold.

Finally, *witnessing the difficult situations* of the recipients themselves engendered uncomfortable interactions for eight participants, as their vulnerabilities and suffering were laid bare:

9. P: knowing that you know that someone who is, especially if they're old, like they're not, they're not going on Zoom, they've not probably got a tonne of people they can be chatting to. They're probably very isolated ‐ to know that like, as soon as I, you know, I cycle off and I've dropped off their stuff they're back to just being totally on their own again. (P10, M, 36, Brighton)

Witnessing such difficult situations also led one participant who had delivered cash to women asylum seekers to an awareness of the asymmetry in power between herself and her recipients:

10. P: I felt there was a strange power dynamic of being quite a young white woman speaking English in an area where there was a lot of, you know, some of the blocks of flats and stuff, it did seem like quite a racist area and there's lots of like Union Jacks up and stuff. And so I think some of the women, I'm not sure if this is me projecting, but it felt like there's like a bit of a strange power dynamic of me being quite intrusive into people's spaces. (P3, F, 26, Manchester)

Here the existing power dynamic between helper and helped was reinforced along racial lines, a dynamic which resonates particularly given the wider context of the UK Covid‐19 mutual aid movement; research indicates that membership of Covid‐19 mutual aid groups was overwhelmingly white and middle‐class (O'Dwyer, 2020a), while the virus itself disproportionately impacted on lower‐income and minority ethnic groups (Public Health England, 2020b). In particular, members of minority ethnic groups may feel marginalized, creating barriers to engagement with healthcare or other services (Public Health England, 2020a). In the present case, the fact that the interaction took place in what appeared to be a racist area accentuated both the power dynamic between “helper” and “helped,” and the participant's self‐perception as being from a potentially hostile outgroup in the eyes of the recipient, creating an uncomfortable feeling of intrusion.

#### Increased sense of engagement in life

Outside of the immediate moment, participants experienced longer‐term impacts to their well‐being via an increased sense of engagement in life. Seven participants referred to a greater feeling of “*purpose*,” which had otherwise been taken away by the disruption of lockdown:

11. I: So do you think that participating in community support has kind of given, o‐over the kind of long period of time, like it has boosted your kind of general mood day to day?
 P: Definitely. Yes. I'd like to feel like I have a purpose in life. And I think that's really important to people's happiness and well‐being if they feel that there's a purpose to what they're doing every day. Your overall well‐being will be better, I think. (P9, F, 38, Brighton)

In the above extract, the participant explicitly connects their desire for purpose to their own well‐being. This relationship between purpose and well‐being was echoed by one participant who lauded mutual aid in more prosaic terms as “something to do,” a welcome distraction from the boredom that lockdown had induced:

12. P: not only did it feel like it was, you know, useful, really rewarding thing to do, but like, there was that, that relief of like, oh, like thank God, I got something to keep me occupied as well. (P10, M, 36, Brighton)

Participation also helped participants to *structure their lives* and to construct a routine alongside other activities:

13. P: It's made me get up earlier, start the day earlier, which I think is very positive, cos then when you're up and you can go out and do more things that you might not have done if you'd still be lying in your bed. (P6, F, 59, Brighton)

Being part of the mutual aid group motivated this participant to go out and “do more things [they] might not have done”: it was not only a purposeful activity in and of itself, but also increased their level of engagement in other areas of their life.

However, participants did not only derive their sense of purpose from the activities considered in isolation. Two of the participants also conceived of their work in broader terms, in relation to its implications for ACORN:

14. P: “definitely not only just like in a, like personal thing but also I knew it was it was helping to, I guess, build the organisation, obviously when, when ACORN's like, like stronger, and the union has got more members, um, as we've had a surge in members during this crisis, I'm sure partly from this campaign that we've been doing, um, we've got more capacity to do stuff like this in the future as well. So, um, that sense as well, it's very, obviously very rewarding helping to build the union as a whole.” (P1, M, 23, Brighton)

The activities were not only meaningful because they served to help others; they also served a larger purpose: to help *build the union*, and recruit more members. Therefore, they were viewed not just as a “personal thing,” but as contributing to the union, the “we” with which the participant identified. This group identification was therefore crucial to seeing building ACORN as a meaningful goal. This was also the case for another participant, for whom the project of building ACORN had helped to rebuild a sense of purpose which had been lost after the failure of a previous project:

15. P: I'd put like, two, almost three years of my life really deeply into a project but I was the person who was the most involved in it. And coming out of that and kind of giving up on it was a real moment of despair. That project has now basically been killed by Coronavirus, which is also a difficult, like, emotional thing for me, but having ACORN has been like amazing for me because it's given me a sense of purpose and a drive where I could have so easily like spiralled into a kind of like aimless depression. (P11, M, 24, Manchester)

#### New or strengthened social relationships

Participants reported a variety of changes to their social relationships, in varying degrees of abstraction, as a result of their participation. On a direct one‐to‐one level, seven of the participants reported *new social bonds*:

16. P: I've lived in this city for such a long time. And sometimes, like, my, my network is fairly small, um, or it's you know in pockets which is to do with places I've worked or people I studied with, so it's nice to meet people who I would never have met otherwise. (P8, F, 28, Brighton)

The participant in extract 16 is discussing new social bonds formed with other food bank volunteers. Another participant developed a relationship with a recipient of aid:

17. P: Yeah, there's definitely one gentleman who's an absolute sweetheart. He's a real regular. The other ones really are ad hoc as and when so, you know, but this guy's twice, twice a week, man. And, uh an interesting, interesting gentleman who's isolated but absolutely coping very well with it. Yeah, we have long chats on the doorstep and stuff. So that's, that's nice. (P6, F, 59, Brighton)

On a group level, the nature of participants’ social relations was moderated by the type of activity with which they were engaged. Thus, none of the participants who only delivered items individually reported any meaningful contact with other volunteers:

18. P: Yeah, I don't know anybody to be honest. I don't know any volunteers. We're all names on a list. (P6, F, 59, Brighton)

In contrast, four of the participants involved in activities where they were interacting with others in the mutual aid group—such as the food bank or organizing volunteers—reported some degree of *camaraderie* with other volunteers: a sense of belonging to a group of people, often from otherwise disparate walks of life, working toward a shared goal:

19. P: it was all everyone chipping in, everyone taking, you know, their time to make sure that these people were, you know, they got their deliveries and got their medicines and stuff. Um, and that yeah, everyone had a part to play, whether they're on the phone or driving around with the deliveries themselves, leafleting or wherever, everyone was, was doing it. (P1, M, 23, Brighton)

In the above extract, the participant's feelings of interconnectedness and positivity toward others, even in the absence of direct contact, is clear. In this case what is important is identification with the category itself, rather than connection to any individual group member. In the case of one participant this sense of camaraderie highlighted something lacking in their ordinary life:

20. P: most of my friends, they they're not interested in volunteering and that, they just don't know why I'm doing it. So I'm like going ‘oh my god, am I the only person who actually wants to do something like this?’ And then you meet all these other people who do as well, and it's just like, nice. Yeah. (P9, F, 38, Brighton)

This extract illustrates the validation that can be provided by being with others who share psychological group membership: “the sense that the beliefs and assumptions that comprise a particular worldview are not idiosyncratic but have a robust basis that is attested to by others” (Hopkins et al., 2019, p. 4). Here, the other volunteers are contrasted favorably with the participant's existing friends, and serve to reduce the participant's feelings of isolation or alienation from others. However, for another participant involved in organizing her own mutual aid group, a lack of shared values produced the opposite effect:

21. P: on a neighbourhood level, I feel like um, I don't know that like, maybe people don't like me that much. Or just think I'm like, tense, or, um trying to push propaganda, which I'm not. But, erm, yeah, so um, I'm not sure how useful I feel in my neighbourhood. (P2, F, 25, Brighton)

The participant goes on to describe a dismissive reception to her advice not to call the police. Here the conflicting viewpoints of the participant and others in her group led to a feeling of division, and of being seen as an unwanted interloper. This in turn decreased the participant's sense of efficacy.

In line with the literature on how groups can contribute to well‐being (Jetten et al., 2014), one participant reported increased expectations of *social support* in the face of potential adversity:

22. P: knowing that this, that these organisations that I was part of, and participating in, like would be there to help me if I was in a similar situation, helping to build that, make sure that, that gives me that kind of sense of, um, I guess, like solidarity among, among renters and working class people who were receiving end of this crisis. (P1, M, 23, Brighton)

This extract chimes with the research by Bowe et al. (2020), which suggests that volunteering can indirectly lead to an increased sense of psychological safety through perceptions of available social support. For this participant this perception was linked to their identity: they viewed themself both as part of an organization (ACORN), and of a wider group of “renters and working class people.” Their actions were thus understood as part of an intragroup exchange based on reciprocity and principles of solidarity.

Finally, at the highest level of abstraction, several participants felt a greater *connection to a community*:

23. P: Just to feel like you're part of a community I think really helps, and my, guess mental map of [Place] has kind of expanded because I know more places, like I wasn't really familiar with [Place], but I know that area now and I know the people, it's just quite nice (P8, F, 28, Brighton)

Here the participant defines community in terms of the people and places they came into contact with. However, definitions of community were not strictly limited to these physically bounded markers. For one participant, their community was conceived of as a virtual world, which existed on the infrastructure through which the network was organized:

24. P: Like just when you look at the spreadsheet, [laughs] it's just like, not to get too deep about a spreadsheet, but you know, all these names, all the people editing it, you know, you can still see that that is a community in and of itself. So I do think it's a way of sort of staying connected to the people around you and, erm, yeah, the place that you live. (P4, F, 27, Brighton)

Importantly, participants’ sense of community was not simply presented to them as a given, but constructed by them out of their own activities, interactions, and narratives regarding the mutual aid network.

#### Greater sense of control

Taking part in the network improved participants’ sense of control, defined as an individual's belief that they can master, shape and control their own life (Keeton et al., 2008). Previous research has shown that an increased sense of control can buffer against a sense of helplessness and psychological distress in the face of adversity (Bennetter et al., 2016), and relieve acute stress symptoms during the Covid‐19 pandemic (Zhou & Yao, 2020). A recurring narrative, deployed by six participants in the present study, was that while the Covid crisis had caused participants to feel helpless, being part of the mutual aid group had helped them to regain a sense of personal *agency*:

25. P: Helped me, it made me feel, um, less, less helpless, I guess. Um, I guess if, if you didn't have organisations, I guess other mutual groups as well but with ACORN we already had the organisational infrastructure to organise this very effectively from the get go. I think without that ‐ would have felt extremely vulnerable. (P1, M, 23, Brighton)

The participant's sense of vulnerability and helplessness was ameliorated by the effectiveness and speed of ACORN's response. Another participant linked their feelings of agency to the immediate responsibilities they were given:

26. P: You know, often if I ever was like, “Oh, yeah, I can do that. If no one else can.” You know, it was always like, “right, well, you're doing it then”, and there's never you know, too much help offered for this group. So I think there's purpose in that, it's made me feel like, er, more agency in, well if I feel something is wrong, what am I personally going to do about it? And also, if I feel like, I have not, like not got purpose or meaning, like what am I going to do about that as well? (P3, F, 26, Manchester)

Mutual aid emphasizes direct involvement by volunteers, without the use of professional intermediaries or hierarchies of delegation (Spade, 2019). This direct involvement caused the participant to view themselves as an agent both with regards to the virus, and in their own personal life.

Control has thus far been understood broadly as control by individuals over their own personal lives. However, four participants also mentioned a sense of control extending beyond this personal level: specifically, a sense of being able to respond in an adversarial manner to those in power, especially with regard to their perceived inadequate response to the virus. It thus boosted participants’ sense of *empowerment*, understood as “confidence in one's ability to challenge existing relations of domination” (Drury & Reicher, 2005, p. 17):

27. P: It's just like, really depressing, obviously to see shitloads of people dying. A lot of them unnecessarily because of negligence from ruling politicians…Um, that's just like deeply saddening and seeing like the countries and the media's response to that has just been, like, I've had to kind of shut off from it a little bit and not not try and engage with it too much, because it's just too like, makes me despair too much, if I think about that too much. Being involved in ACORN is a good antidote to that because I genuinely feel like we're building a force that can challenge that kind of thing. (P11, M, 24, Manchester)

This participant is a coordinator for ACORN, and therefore holds a strong social identity as a member of the group. They connect the high number of deaths from the virus to “negligence from ruling politicians”: therefore, their aim is not simply to ameliorate the immediate consequences of the virus, but to bring about social change to tackle an underlying problem. Here the prior concept of “building the union” becomes relevant—by growing the union to become “a force,” the participant will be able to challenge those in power more effectively, and insofar as the mutual aid movement contributes to that, it becomes a source of empowerment.

Another participant identified the work itself as empowering:

28. P: I think just on a personal level, even if I'm not doing, you know, a huge amount of work, I just think it makes me feel less like I'm sort of passive ‐ like, Oh God, I don't know what's going to happen, and sort of my health or my wellbeing really depends on what this virus and what the government decides. I think that that contributed to quite a lot of anxiety at the beginning of the pandemic ‐ was just feeling like I didn't have any control. So I think doing small things like this it feels like, you know, I'm doing something to help those sorts of problems that can make me feel so worried. And I think that that's maybe sort of the biggest boost in a selfish way often from activism is just it gives you a bit more power and control, and lets you do some good things in the world (P4, F, 27, Brighton)

This extract is interesting for two reasons. First, the participant links the anxiety they felt from the pandemic to the feeling that they “didn't have any control.” Second, their participation in the mutual aid group is framed in explicitly political terms: “activism,” rather than, say, “volunteering.” In this context participation in a mutual aid group is a reclamation of political agency: creating a solution to the government's inadequate response.

Finally, for one participant empowerment was not only an outcome of participation in the group, but a characteristic of the group itself:

29. P: actually demonstrating to people, um actually materially improving people's lives through, not charity, through like organisation, making sure we're all organised together, demonstrates this like, demonstrates, like, what power you have when you do, when you are organised together and not atomized in these individual, like not part of the Union, all atomized and all just like transacting, you know, in this like, kind of, transactional kind of society that people are used to (P1, M, 23, Brighton)

The efficacy of the mutual aid network, which contrasts with the “transactional” practices of existing society, serves at once as a realization of the participant's social identity; a demonstration of group power; and a model of a possible future world that validates the legitimacy of their political beliefs. The above therefore serves as an example of collective self‐objectification (Drury et al., 2015)—that is, action that actualizes participants’ social identity over against the power of dominant groups.

### Part 2: The role of political framing

As noted earlier, mutual aid may be viewed either politically or apolitically. In the present case, while all four interviewees who were already ACORN members viewed their participation as political, only two of the remaining seven participants viewed their participation as such (see Table 2). One ACORN member noted that the Coronavirus Community Support campaign had attracted many participants outside of ACORN's core membership precisely because of this:

30. P: Um, the, the like, the kind of action that we take, like, protesting, like picketing outside letting agencies, um, like banners and placards and T shirts and chants, um language, that kind of language that we use that goes with it, um, puts some people off. It doesn't speak to them, because of the militan ‐ because of like the militancy in it. Um, um, and, but our coronavirus, community support work, the mutual aid work, um, it doesn't have that same, um it doesn't have that same element to it. It's not about bringing people together to fight a target, it's bringing people together to support each other, which is a much easier sell, um, in the ideological climate of 21st century Great Britain. It like plays on popular ideas of like niceness and looking out for each other, which are like much more widely felt than sentiments of, um, class struggle. Um, so it, so that's why I think it appealed to a much wider audience and brought in a lot more people. (P11, M, 24, Manchester)

Therefore, we may broadly divide the participants of this study into two groups: ACORN members with a preexisting politicized collective identity and individuals who were not previously involved with ACORN, but had joined for the Covid campaign and mostly viewed their participation as apolitical. What are the implications of these differing identities on the effects of participation?

It is notable that all four participants who were ACORN members cited empowerment as an experiential outcome; none of the non‐ACORN members did so. Additionally, two ACORN members cited building the union as providing meaning; again, none of the non‐ACORN members did so. This is unsurprising, because both processes specifically relate to the political identity of participants: empowerment requires participants to view themselves, at least in an incipient sense, as opposing existing structures; building ACORN cannot be seen as a meaningful goal without some level of identification with ACORN. Furthermore, the two processes are connected: building the union was seen as strengthening the group, leading to greater feelings of empowerment. Additionally, the only participant who reported feelings of social support was an ACORN member. While perceptions of social support need not be restricted to those with a political identity, they are based on a sense of shared identity (Drury et al., 2019); here, this consisted of the participant's identity as an ACORN member. Moreover, the participant's perception of social support was specifically driven by the political perspective through which they viewed mutual aid, as based on principles of reciprocity and solidarity. However, while the lack of a politicized group identity inhibited these processes, the reverse did not apply; the ACORN members nonetheless accessed the other benefits of participation. Therefore, participants who viewed their participation through the lens of a political identity experienced additional benefits.

On its own, however, this analysis is insufficient. Previous work has conceptualized “empowerment” not only as an outcome of any given collective action, but as a dynamic process of transformation, which is at least partly explicable through the actions of individuals in intergroup contexts which create an emergent collective identity (Drury & Reicher, 2009). Participants’ identities should therefore be understood as malleable, rather than predetermined as “political” or “apolitical.” Wein (2020) finds that almost 20% of mutual aid participants intend to pay more attention to politics after the pandemic, while 83% of participants intend to take some form of political action in the coming year. However, this in itself does not provide reason to believe that change flows from participation in mutual aid, rather than the wider pandemic scenario itself. In what follows we present a hypothesis for how participation in mutual aid may have an independent politicizing effect, using the interview data to identify two complementary processes by which this may be achieved.

The first process relates to participants’ emotional experiences. While all 11 participants experienced some sort of positive emotion during participation, 8 of them also experienced negative emotions due to *witnessing the difficult situations* of the recipients:

31. P: I guess because I felt so uncomfortable a lot of the time doing it, and it was, yeah, it felt like quite a humiliating experience for the women and quite like, yeah, an uncomfortable one for me. Um, because of that it kind of felt like, I don't know, it made me feel more aware of some of the difficulties that these women are in and I, like, felt more engaged with that. And so I guess the nature of it being a bit of an awkward experience has made me feel like, right, well, this doesn't, it seemed like unjust as a situation, it kind of made me feel like “Right, well, what am I doing about this? How else can I support people in my community who are experiencing this?” (P3, F, 26, Manchester)

Here the discomfort of the experience is part of recognizing the situation as wrong: this feeling further highlighted the difficulties faced by those in the participant's community, and provided motivation to support them. Four participants reported greater awareness of disadvantaged groups:

32. P: and it, and I just, opened my eyes to how much poverty actually there is ‐ I didn't realise all these poor people really couldn't afford the meal, because it's not just Covid. (P9, F, 38, Brighton)

This statement indicates a newfound awareness of issues extending outside of Covid‐19, which the pandemic brought to light. Yet while this might increase intergroup awareness, politicization is not guaranteed. After all, we may witness and condemn suffering without identifying its cause as political. As Simon and Klandermans (2001) note, an adversarial attribution must also be generated. Hence the requirement for a second, interlinked process: *perceived inadequacy of government response*. By questioning why recipients relied on the mutual aid network in the first place, participants were moved to question the government's response:

33. I: And has participating in it led to any change in your beliefs about how the government should be responding to the virus?
 P: Um, I mean, yeah, it has crossed my mind that like if things like ACORN weren't running, like what would, like what would these people have had instead, like? … what the heck would have happened to them otherwise? (P10, M, 36, Brighton)

This line of thought is a logical extension of that in *making a difference* (above): what would have happened if the participant had not stepped in, and how did recipients come to such a desperate situation in the first place? Nine participants viewed the government response as inadequate. One participant utilized the high demand to formulate an explicit criticism of the government:

34. P: I think it's highlighted the gap and how vulnerable some people are. So yeah, and it also made me think that the government was extremely slow in taking action…I know that the government is sending, uh, food parcels out but they're clearly not meeting everybody because we wouldn't have such high demand otherwise. (P8, F, 28, Brighton)

The initial discomfort is the trigger to view the situation as wrong; subsequent perceived inadequacy of government response is a step toward viewing it as unjust. Together, these two processes may engender a political grievance. Furthermore, if this grievance is then perceived as shared with other members of the mutual aid group via processes such as validation, it may form the basis of a politicized collective identity (Klandermans, 2014). Therefore, while witnessing suffering can be unpleasant, its potential to change the worldview of participants may paradoxically unlock additional longer‐term benefits for well‐being—through politicization, which facilitates more positive experiences of participation in the group than for nonpolitical participants. A possible corollary of this is that the positive feelings experienced during participation may, if they serve to obfuscate the negative features of the wider situation, inhibit politicization. The conflicting experiential processes at play in the immediate helping scenario thus mirror the tension between the societal narratives ascribed to mutual aid: on one hand, a feel‐good activity in which those in need receive the help they require through community volunteers; on another, a difficult political project in which groups on uneven footing take responsibility for one another in an attempt to forge meaningful bonds of solidarity. One participant captured this contradiction evocatively:

35. Erm, I dunno, so I saw on ACORN, a video that someone had taken after they've done a similar, er, drop‐off, for the same groups, so the same thing that I was doing… some of the videos that I've seen, have been like, “Oh, you know, I just dropped these off, these women are in destitution. They don't have understand, like they don't have, understand what else they can do. And that's why they've come to ACORN. And ACORN's so brilliant because we've helped these women and without ACORN, where would they be?” And it just didn't, it was, felt like a different reaction to how I instinctively felt about it, which was that like, it was like a sense of pride, which I understand. And I think it's really good maybe to recruit new people and stuff is to be like, we are so great at tackling this injustice. Whereas I guess the way that I felt it was more like, oh, there's this horrible injustice happening. And actually, I don't know, I guess I can maybe do this, but it doesn't fix the problem. And I feel, I don't know. It just made me feel uncomfortable with my position in society rather than making me feel proud about my position as someone trying to help it. (P3, F, 26, Manchester)

## DISCUSSION

Active public engagement and participation is an essential part of a public health campaign in relation to disease prevention in epidemics (Costello, 2018). In particular, public participation is required to support those who need to self‐isolate or who are dependent in other ways. The rise of Covid‐19 mutual aid and other community support groups meets this need. Yet such participation takes a toll on the volunteers themselves. The phenomenon of social support deterioration following disasters, as “disaster communities” run out of energy and resources, is well established in the literature (Kaniasty & Norris, 1999). Moreover, unlike some other forms of group activity and other kinds of volunteering, activism can lead to burnout (Chen & Gorski, 2015). There is a practical need to understand the factors that mitigate against this decline and burn out, so that organizers of mutual aid groups have more strategies to sustain and motivate their members over the duration of a pandemic that could last for many months.

In examining the possible well‐being effects of participation in Covid‐19 mutual aid groups, this study has sought to contribute to theory as well as address an important practical question. We have examined the relevance of the “social cure” approach (Haslam et al., 2018) for a new domain (Covid‐19 mutual aid groups) and explored the moderating role of type of group identity (political vs. nonpolitical) on well‐being outcomes and processes.

All participants reported some form of positive impact to their well‐being as a result of their participation, which could be divided into four categories: positive emotional experiences, increased sense of engagement in life, new or improved social relationships, and greater sense of control. However, interviewees also reported some negative emotional experiences.

While this division of outcomes appears to neatly match the social cure mechanisms of connection, meaning, support, and agency (Haslam et al., 2018), some participants’ experiences were more clearly linked to identity processes than others. For example, participants often considered their work meaningful because it made a difference to others; over time, this provided a sense of purpose and an activity with which to structure their time. Participants also felt more agency by taking action against the virus. While being part of the group made these experiences possible, in neither case did participants explicitly reference their social identities. Furthermore, participants’ new social bonds were often arguably better conceptualized as interpersonal friendships than shared social identity (Fehr & Harasymchuk, 2018).

For other well‐being consequences, the role of social identity was clearer and indeed was different according to the content of that identity. Those volunteers with a politicized collective identity as ACORN members emphasized their feelings of empowerment as a result of this identity, while those who possessed a more apolitical shared identity linked this to feelings of connectedness and camaraderie. This replicates the findings of Hatzidimitriadou (2002), who compared members of mental health mutual‐aid groups emphasizing either personal or social change: members of the former reported greater sharing of feelings, while members of the latter reported a greater sense of control and feelings of power. Thus participation in mutual aid may empower communities, allowing them to challenge the root causes of inequality (Spade, 2020); however, the present findings suggest that this is best achieved if participation is seen through a political lens. Thus, while the present study replicates the finding by Stevenson and colleagues that volunteering can enhance well‐being through a social cure process (Bowe et al., 2020; Gray & Stevenson, 2020), it goes beyond this work by showing that these processes can apply even in conditions of pandemic, where volunteers are potentially vulnerable themselves.

For some participants, the pandemic scenario and requirements for social distancing undermined the formation of shared social identities—their work generally involved delivering food or prescriptions individually to recipients, who were often vulnerable in a way that precluded prolonged contact and connection. However, other participants created a sense of shared social identity in several ways. The most direct method was through regular contact with other volunteers, such as the food bank. However, this was not the only method: participants were also able to construct a (new) sense of groupness via their symbolic understanding of their own actions, relationships with people and places, and their perceptions of the network. The present study therefore provides further evidence that volunteering increases a sense of community belonging (Theurer & Wister, 2010), but also sheds light on the variety of ways a “psychological sense of community” (Burton et al., 2019) may be generated.

While we believe the “social cure” provides a valuable theoretical framework for understanding and interpreting our findings, these data do not necessarily rule out other theoretical approaches. For example, some our findings could be compatible with Self‐Determination Theory (Deci & Ryan, 2000), which posits that humans have three innate psychological needs: for autonomy, competence, and affiliation. However, the advantage of the social identity framework is that it offers a way of understanding variations in experiences of efficacy and so on as linked to variations in identity (rather than being tied to an individual essence). Thus for example one experiences greater efficacy not only when the group achieves its goal but also when one identifies with the group. In our interview data, those with a “political” identity experienced empowerment more than those without such an identity.

### Limitations and future directions

This study has a number of limitations. The sample was generally middle class, which appears to reflect the general demographic trend for Covid‐19 mutual aid groups in the United Kingdom (Felici, 2020). Therefore, while this study may be applicable to many mutual aid groups, it remains unclear whether the underlying processes can be generalized to a wider population. For example, it is possible that the observed psychological benefits of mutual aid only apply once more “basic” physiological and protective needs are fulfilled (Maslow, 1970). With this in mind, whether members of disadvantaged communities experiencing material deprivation can be empowered through mutual aid requires further study. Nonetheless, we believe it is still worthwhile to investigate how psychological benefits can be attained, even if our findings are currently limited to conditions of relative material security.

Second, it is unclear whether viewing the mutual aid group through a political lens increases well‐being overall compared to an apolitical lens, given that activists may hold more “macroworries” (Boehnke & Wong, 2011), which are harder to address via mutual aid, and are susceptible to burnout (Gorski et al., 2019). Therefore, our findings should be viewed as complementary to future quantitative work assessing the relative benefits of different forms of social and political identification. For example, recent findings by O'Dwyer (2020b) indicate that perceptions of mutual aid as political positively moderated participants’ feelings of perceived support, collective efficacy and coping self‐efficacy.

Third, there are two potential confounds with the “political” identity. ACORN members volunteered on average for a greater amount of time when compared to non‐ACORN members (an average of 14.63 h per week compared to 7.34 h per week). In addition, ACORN members were both more likely than nonmembers to possess a prior sense of “groupness.” With regard to the first possible confound, it is certainly possible that a greater level of participation would have a greater effect, not only on positive measures but also negative measures, such as stress. However, it is worth noting that of the three participants who were not ACORN members and volunteered large amounts of time (P8, P9, and P10) none reported experiencing empowerment, building the union as a source of engagement, or social support, despite experiencing a large range of other benefits. By contrast, the ACORN members with relatively lower levels of participation (P1 and P4) both reported empowerment, while P1 also reported building the union and social support. This suggests that a large amount of participation was not by itself enough to access these benefits. With regards to the second possible confound, while we accept that there was no preexisting shared identity which was apolitical, for four participants who were not ACORN members a new group identity did emerge (P3, P8, P9, P10), either as a member of the community or through camaraderie with other volunteers. Again, these participants reported other benefits related to this group identity, but not those benefits which we identify in our study as appearing to stem from the distinctively political identity of being an ACORN member.

In terms of future directions, the hypothesized process by which participants in mutual aid may become politicized requires further rigorous examination. It sketches out in further detail a suggestion which has already been made by other scholars: that the rise of mutual aid may rebuild bonds of intergroup solidarity, which have eroded under successive neoliberal regimes (McGregor, 2020; O'Dwyer, 2020a). A large volume of “politicization” research examines it in the context of explicitly confrontational activity such as protests (e.g., Drury & Reicher, 2000). However, this focus on conflict neglects to examine other forms of political activity, such as prefigurative projects (e.g., Biddau et al., 2016) as well as less explicitly “political” activities, which can nonetheless politicize, such as contact with disadvantaged groups (e.g., Monforte, 2019). This research therefore serves as a contribution to the existing body of psychological research, which moves beyond conventional forms of “activist” behavior to the “everyday” processes and contexts in which political grievances and identities may be formed (Fernandes‐Jesus et al., 2018; Smith et al., 2015).

This study ties together two growing fields of literature: disaster studies and the social cure. By doing so, it provides a theoretical framework and suggestive complementary evidence to understand the observation, made by Fritz (1996) and others, that disasters are associated with counterintuitively mentally healthy populations—at least among those giving and perceiving support (Kaniasty & Norris, 1999). Moreover, longitudinal data on the effects of Covid‐19 on the mental health of the UK population challenges the idea that Covid‐19 led to an across‐the‐board “tsunami” of mental health problems; rather, the impact of the pandemic was heterogeneous, with individuals exhibiting different longitudinal profiles in their mental health (Shevlin et al, 2021). While the majority of the population have been part of a “resilient” class which showed little‐to‐no psychological distress, others have exhibited a range of different trajectories, including those who have deteriorated and those who have exhibited considerable improvement in their mental health. Compared to members of the “resilient” class, those who exhibited some level of mental distress were generally associated with higher loneliness, external locus of control, and death anxiety. Therefore, further research should investigate whether participation in mutual aid, by leading to a greater sense of control and combating loneliness, could promote greater psychological “resilience” during disasters.

Finally, our hypothesis serves as a reminder of the value of negative affect. “Hedonic” conceptualizations of well‐being overwhelmingly emphasize positive affect while minimizing negative affect (Fredrickson & Losada, 2005). In contrast, “eudaimonic” conceptualizations, based on Aristotle (ca. 350 B.C.E./1925), operationalize well‐being in terms of indicators such as meaning in life or self‐realization (Waterman, 1993). But this is an odd reading of Aristotle, who conceptualized eudaimonia primarily as the development and instantiation of “virtues,” understood as dispositions to feel and act in “right” ways in given contexts (Roberts, 1989). Within this schema, negative emotions such as fear and anger can be felt “both too much and too little” (Aristotle, 1925, 1106b). In this schema, negative affect in the face of injustice may even embody the virtue of “righteous indignation” (Nicolas, 2017). Similarly, Atkinson (2013) notes that people generally do not endorse an “uncritical” happiness founded on deficient cognitive or affective states, such as lack of care for others. Therefore, the study encourages a conception of well‐being as a combination of affective, cognitive and behavioral dispositions, where negative emotions in given contexts are valued not only for any epiphenomenal consequences, but are themselves constitutive of well‐being.

### Practical implications

This research forms the basis for two practical suggestions. For authorities dealing with the Covid‐19 pandemic, this research ties into work on community resilience (Norris et al., 2008; Plough et al., 2013)—defined in (the United Kingdom) policy as “the public … empowered to harness local resources and expertise to help themselves and their communities to prepare, respond and recover from disruptive challenges, in a way that complements the activity of … emergency responders” (Cabinet Office, 2019). The authorities have relied on public involvement, not simply in adherence to the public health behavioral guidance (hand‐washing, distancing, wearing masks) but crucially in a more proactive role—in supporting those in self‐isolation, running foodbanks, and acting as “community champions” to disseminate public health information (SPI‐B, 2020b). But while many authorities may be tempted to reach out to Covid‐19 mutual aid groups as “third‐sector” organizations, and some have now constituted themselves as charities to work more closely with the local authority and obtain funding, the more politically oriented groups outright reject collaboration with state structures. Furthermore, attempts to co‐opt such groups or mitigate their political character may backfire by bringing to the fore volunteers’ conflicting viewpoints, undermining shared identity (Drury et al., 2019), and disempowering more politically minded participants. Authorities should therefore seek to provide support via a “facilitative” approach (Tiratelli & Kaye, 2020), for example through signposting of services, proactively connecting existing groups, or provision of infrastructure, while avoiding excessive micromanagement.

Our findings also support adoption of mutual aid as part of the strategic repertoire of movement organizations (Spade, 2020). First, mutual aid has the potential to empower members who already possess a political identity. Drury et al. (2005) found that collective self‐objectification was amongst the most commonly cited factors by activists in explaining their experiences of empowerment. It is hard to imagine better praxis in this sense than mutual aid, insofar as it is quite literally the “reali[sation] in the here and now aspects of a world that does not yet exist” (Drury & Reicher, 2009, p. 722). Second, mutual aid can mobilize wider sections of the population who are not usually politically engaged, both by playing on popular norms, and by offering opportunities to enhance their well‐being. Moreover, if our hypothesis is correct, by fostering contact with disadvantaged groups, mutual aid can facilitate the emergence of new political identities. All of these advantages are neatly illustrated by the fact that ACORN experienced record growth throughout the Covid‐19 “lockdown” in the United Kingdom, and groups are now able to carry out their eviction resistance work with a significantly expanded base of activists (ACORN UK, 2020b). In this sense, the pandemic has been an opportunity for “activist” community groups to demonstrate their value to many more people.

## CONCLUSIONS

Covid‐19 mutual aid groups have served two crucial functions in the pandemic. First, they have met the emotional and material needs of those requiring support. Specifically, the challenges of self‐isolation would be impossible without the support provided by mutual aid groups and others. Second, our analysis shows these groups have served to enhance the well‐being of the volunteers themselves. Our findings on the well‐being effects of providing support are probably not unique to mutual aid groups. Rather, our argument is that participation in mutual aid during disasters appears to be one context in which social cure processes are instantiated. In addition, these processes differed based on the different activities that the participants engaged in; those who engaged in more individualistic ways reaped benefits which were unrelated to their social identity, while those who possessed a shared identity were able to access benefits such as feelings of connection, camaraderie and empowerment. These differing experiences provide a level of comparison and contextualization within the study, and therefore strengthen the case for the role of group identification—as suggested by the “social cure approach.”

In the short‐term, mutual aid groups face the challenge of sustaining themselves, with demand increasing when the United Kingdom entered a second wave in Autumn 2020 (SPI‐B, 2020b). The present research suggests that sustained volunteer engagement may be encouraged through the benefits to well‐being it provides, and that this is especially the case if participants possess a shared (“political”) social identity. In the long term, however, mutual aid groups with a more radical orientation face a more subtle challenge in reconciling two goals: on one hand, cultivating a sense of shared social identity between members; on the other, expanding conceptions of that identity. This is particularly important given that membership tends to correlate with socioeconomic status. This could either lead to a situation where groups are strongly unified based on existing similarities and form strong norms of reciprocity, in which case those who are deprived do not receive the help they need; or a situation suspiciously similar to “charity,” whereby better‐off members offer one‐sided help to those unable to reciprocate. More generally, there is a tension between framing those being helped as part of the “in‐group,” and highlighting the material inequalities and differences between helper and helped in a way that will contribute to a deeper political understanding. Furthermore, as the network expands it will necessarily have to accommodate groups with disparate sets of grievances. Of course, this is far from impossible, and change throughout history has occurred when diverse groups recognize their commonalities and stand together. As our findings demonstrate, people can do a good job of creating a shared identity by themselves. But their understandings of who that identity encompasses, and what its potentialities are, are not always the same. To forge a shared understanding that can accommodate these identities, and unify them in a collective claim for liberation, is both the challenge, and the promise, of the slogan: solidarity, not charity.

## CONFLICT OF INTEREST

We have no conflicts of interest to declare.

## AUTHOR CONTRIBUTIONS

GM was responsible for study design, data collection, and analysis, and writing. JD was responsible for supervision, writing, and manuscript preparation. MFJ and EN were responsible for writing. All authors approved the final manuscript for submission.^1^

### OPEN RESEARCH BADGES

This article has earned Open Data and Open Materials badges. The research in this paper is not preregistered, but the authors have made available all data, analytic methods (e.g., code), and study materials at the Open Science Framework (https://osf.io/3z5u2/?view_only=696a14f62b1b4130826c4fe0e0934918).

## Data Availability

The redacted transcripts and interview schedule are available at https://osf.io/3z5u2/?view_only=696a14f62b1b4130826c4fe0e0934918. All interview questions have been reported. All transcripts are shared, except for that of two participants who did not consent to sharing their data. This project was not preregistered.

## Footnotes

1 The redacted transcripts and interview schedule are available at https://osf.io/3z5u2/?view_only=696a14f62b1b4130826c4fe0e0934918. All interview questions have been reported. All transcripts are shared, except for that of two participants who did not consent to sharing their data. This project was not preregistered.

## References

[asap12275-bib-0001] ACORN . (2020) ACORN Coronavirus Community Support. ACORN—Union for the Community. Available from: https://dev.acorncommunities.org.uk/2020/corona/

[asap12275-bib-0002] ACORN UK. (2020a) ACORN UK on Twitter: ‘Thanks to everybody who has volunteered with ACORN Coronavirus Community Support—We've now helped over 1000 households across the country. https://t.co/5eKSJdO3hf Also shout out to all NHS staff/volunteers & everyone else who has helped people in need during the crisis. https://t.co/Gb6SUb3Sne_/Twitter. Twitter. https://twitter.com/home, https://twitter.com/ACORN_tweets/status/1247598557823152129

[asap12275-bib-0003] ACORN UK. (2020b, July 9) We recorded 8.5% growth in June, another record following several months of record growth. Over a quarter million renters are facing eviction in August. Our union is ready to fight for our members & our communities. Join us—https://acorntheunion.org.uk/join/. Facebook. https://www.facebook.com/acorntheunion/posts/3359668810760811

[asap12275-bib-0004] Addley, E. (2020, June 5) Making up with the Joneses: How COVID‐19 has brought neighbours closer. Available from: https://www.theguardian.com/world/2020/jun/05/neighbourliness‐to‐the‐fore‐its‐been‐the‐highlight‐of‐our‐lockdown

[asap12275-bib-0005] Auf der Heide, E. (2004) Common misconceptions about disasters: Panic, the ‘Disaster Syndrome,’ and Looting. In: O'Leary , M. (Ed.), The first 72 hours: A community approach to disaster preparedness. Lincoln, NE: iUniverse Publishing.

[asap12275-bib-0006] Aristotle . (1925) Nicomachean ethics. Book II (W.D. Ross Trans). The Internet Classic Archive. http://classics.mit.edu/Aristotle/nicomachaen.2.ii.html (Original work published 350 B.C.E)

[asap12275-bib-0007] Atkinson, S. (2013) Beyond components of wellbeing: The effects of relational and situated assemblage. Topoi, 32(2), 137–144. 10.1007/s11245-013-9164-0 25821285 PMC4371813

[asap12275-bib-0008] Barton, A.H. (1969) Communities in disaster: A sociological analysis of collective stress situations. New York, NY: Doubleday.

[asap12275-bib-0009] Bennetter, K.E. , Clench‐Aas, J. & Raanaas, R.K. (2016) Sense of mastery as mediator buffering psychological distress among people with diabetes. Journal of Diabetes and Its Complications, 30(5), 839–844. 10.1016/j.jdiacomp.2016.03.022 27085604

[asap12275-bib-0010] Biddau, F. , Armenti, A. & Cottone, P. (2016) Socio‐psychological aspects of grassroots participation in the transition movement: An Italian case study. Journal of Social and Political Psychology, 4(1), 142–165. 10.5964/jspp.v4i1.518

[asap12275-bib-0011] Big Door Brigade . (2020) What is mutual aid?—Big door brigade. Available from: https://bigdoorbrigade.com/what‐is‐mutual‐aid/

[asap12275-bib-0012] Boehnke, K. & Wong, B. (2011) Adolescent political activism and long‐term happiness: A 21‐year longitudinal study on the development of micro‐ and macrosocial worries. Personality and Social Psychology Bulletin, 37(3), 435–447. 10.1177/0146167210397553 21307181

[asap12275-bib-0013] Bowe, M. , Gray, D. , Stevenson, C. , McNamara, N. , Wakefield, J. , Kellezi, B. , et al. (2020) A social cure in the community: A mixed‐method exploration of the role of social identity in the experiences and well‐being of community volunteers. European Journal of Social Psychology, 10.1002/ejsp.2706

[asap12275-bib-0014] Braun, V. & Clarke, V. (2013) Successful qualitative research: A practical guide for beginners. London, UK: Sage.

[asap12275-bib-0015] Braun, V. & Clarke, V. (2006) Using thematic analysis in psychology. Qualitative Research in Psychology, 3(2), 77–101.

[asap12275-bib-0016] Burton, M. , Kagan, C. , Duckett, P. , Lawthom, R. & Siddiquee, A. (2019) Critical community psychology: Critical action and social change (2nd ed.). London: Routledge.

[asap12275-bib-0017] Cabinet Office . (2019) Community resilience development framework. Available from: https://www.gov.uk/government/publications/community‐resilience‐development‐framework

[asap12275-bib-0018] Cant, C. (2018) Taking what's ours: An ACORN inquiry. Notes from Below. Available from: https://notesfrombelow.org/article/taking‐whats‐ours‐an‐acorn‐inquiry

[asap12275-bib-0019] Chen, C.W. & Gorski, P.C. (2015) Burnout in social justice and human rights activists: Symptoms, causes and implications. Journal of Human Rights Practice, 7(3), 366–390. 10.1093/jhuman/huv011

[asap12275-bib-0020] Cherniss, C. (1972) Personality and ideology: A personological study of women's liberation. Psychiatry: Journal for the Study of Interpersonal Processes, 35(2), 109–125. 10.1080/00332747.1972.11023706 5024902

[asap12275-bib-0021] Clarke, L. (2002) Panic: Myth or reality? Contexts, 1(3), 21–26.

[asap12275-bib-0022] COVID‐19 Mutual Aid UK . (2021) Find your local group—COVID‐19 mutual aid. Available from: https://covidmutualaid.org/local‐groups/

[asap12275-bib-0023] Costello, A. (2018, May 20). A social vaccine for Ebola. A lesson for the Democratic Republic of Congo. Available from: https://www.anthonycostello.net/2018/05/20/a‐social‐vaccine‐for‐ebola‐a‐lesson‐for‐the‐democratic‐republic‐of‐congo/

[asap12275-bib-0024] Cruwys, T. , Dingle, G.A. , Haslam, C. , Haslam, S.A. , Jetten, J. & Morton, T.A. (2013) Social group memberships protect against future depression, alleviate depression symptoms and prevent depression relapse. Social Science & Medicine, 98(1982), 179–186. 10.1016/j.socscimed.2013.09.013 24331897

[asap12275-bib-0025] Deci, E.L. & Ryan, R.M. (2000) The "what" and "why" of goal pursuits: Human needs and the self‐determination of behavior. Psychological Inquiry, 11(4), 227–268. DOI: 10.1207/S15327965PLI1104_01

[asap12275-bib-0026] Dhillon, A.S. (2020) The politics of Covid‐19: The frictions and promises of mutual aid. *Red Pepp*er. Available from: https://www.redpepper.org.uk/the‐politics‐of‐covid‐19‐the‐frictions‐and‐promises‐of‐mutual‐aid/

[asap12275-bib-0027] Dodson, L. & Schmalzbauer, L. (2005) Poor mothers and habits of hiding: Participatory methods in poverty research. Journal of Marriage and Family, 67(4), 949–959.

[asap12275-bib-0028] Drury, J. (2018) The role of social identity processes in mass emergency behaviour: An integrative review. European Review of Social Psychology, 29(1), 38–81. 10.1080/10463283.2018.1471948

[asap12275-bib-0029] Drury, J. , Carter, H. , Cocking, C. , Ntontis, E. , Tekin Guven, S. & Amlôt, R. (2019) Facilitating collective psychosocial resilience in the public in emergencies: Twelve recommendations based on the social identity approach. Frontiers in Public Health, 7, 141. 10.3389/fpubh.2019.00141 31214561 PMC6558061

[asap12275-bib-0030] Drury, J. , Cocking, C. , Beale, J. , Hanson, C. & Rapley, F. (2005) The phenomenology of empowerment in collective action. British Journal of Social Psychology, 44(3), 309–328. 10.1348/014466604X18523 16238842

[asap12275-bib-0031] Drury, J. , Evripidou, A. & Van Zomeren, M. (2015) Empowerment: The intersection of identity and power in collective action. In: Sindic , D. , Barreto , M. & Costa‐Lopes , R. (Eds.), Power and identity. London: Psychology Press, pp. 94–116.

[asap12275-bib-0032] Drury, J. & Reicher, S. (2000) Collective action and psychological change: The emergence of new social identities. British Journal of Social Psychology, 39(4), 579–604. 10.1348/014466600164642 11190686

[asap12275-bib-0033] Drury, J. & Reicher, S. (2005) Explaining enduring empowerment: A comparative study of collective action and psychological outcomes. European Journal of Social Psychology, 35(1), 35–58. 10.1002/ejsp.231

[asap12275-bib-0034] Drury, J. & Reicher, S. (2009) Collective psychological empowerment as a model of social change: Researching crowds and power. Journal of Social Issues, 65(4), 707–725. 10.1111/j.1540-4560.2009.01622.x

[asap12275-bib-0035] Dunn, P. , Allen, L. , Cameron, G. & Alderwick, H. (2020) COVID‐19 policy tracker. The Health Foundation. Available from: https://www.health.org.uk/news‐and‐comment/charts‐and‐infographics/covid‐19‐policy‐tracker

[asap12275-bib-0036] Dwyer, P.C. , Chang, Y.‐P. , Hannay, J. & Algoe, S.B. (2019) When does activism benefit well‐being? Evidence from a longitudinal study of Clinton voters in the 2016 U.S. presidential election. Plos One, 14(9), e0221754. 10.1371/journal.pone.0221754 31487304 PMC6728069

[asap12275-bib-0037] Fredrickson, B.L. & Losada, M.F. (2005) Positive affect and the complex dynamics of human flourishing. The American Psychologist, 60(7), 678–686. 10.1037/0003-066X.60.7.678 16221001 PMC3126111

[asap12275-bib-0038] Fehr, B. & Harasymchuk, C. (2018) The role of friendships in well‐being. In: Maddux, J.E. , Subjective well‐being and life satisfaction. 1st, Routledge/Taylor & Francis Group, pp. 103–128.

[asap12275-bib-0039] Felici, M. (2020) Social capital and the response to Covid‐19. Bennett Institute. Available from: https://www.bennettinstitute.cam.ac.uk/blog/social‐capital‐and‐response‐covid‐19/

[asap12275-bib-0040] Fernandes‐Jesus, M. , Lima, M.L. & Sabucedo, J.M. (2018) Changing identities to change the world: Identity motives in lifestyle politics and its link to collective action. Political Psychology, 39(5), 1031–1047. 10.1111/pops.12473

[asap12275-bib-0041] Foster, M. (2014) Everyday confrontation of discrimination: The well‐being costs and benefits to women over time. International Journal of Psychological Studies, 5. 10.5539/ijps.v5n3p135

[asap12275-bib-0042] Fritz, C.E. (1996) Disasters and mental health: Therapeutic principles drawn from disaster studies. Disaster Research Center.

[asap12275-bib-0043] Fritz, C. & Williams, H. (1957) The human being in disasters: A research perspective. The Annals of the American Academy of Political and Social Science, 309 (1), 42–51

[asap12275-bib-0044] Gorski, P. , Lopresti‐Goodman, S. & Rising, D. (2019) Nobody's paying me to cry”: The causes of activist burnout in United States animal rights activists. Social Movement Studies, 18(3), 364–380. 10.1080/14742837.2018.1561260

[asap12275-bib-0045] Gray, D. & Stevenson, C. (2020) How can ‘we’ help? Exploring the role of shared social identity in the experiences and benefits of volunteering. Journal of Community & Applied Social Psychology, 30(4), 341–353. 10.1002/casp.2448

[asap12275-bib-0046] Grayson, D. (2020, April 28) Mutual aid and radical neighbourliness. Lawrence & Wishart. Available from: https://www.lwbooks.co.uk/blog/mutual‐aid‐and‐radical‐neighbourliness

[asap12275-bib-0047] Grimm, A. , Hulse, L. , Preiss, M. & Schmidt, S. (2014) Behavioural, emotional, and cognitive responses in European disasters: Results of survivor interviews. Disasters, 38, 62–83. 10.1111/disa.12034 24325239

[asap12275-bib-0048] Guest, G. , Bunce, A. & Johnson, L. (2006) How many interviews are enough? An experiment with data saturation and variability. Field Methods, 18(1), 59–82. 10.1177/1525822X05279903

[asap12275-bib-0049] Haslam, C. , Haslam, S. , Jetten, J. , Bevins, A. , Ravenscroft, S. & Tonks, J. (2010) The social treatment: The benefits of group interventions in residential care settings. Psychology and Aging, 25, 157–167. 10.1037/a0018256 20230136

[asap12275-bib-0050] Haslam, C. , Holme, A. , Haslam, S. , Iyer, A. , Jetten, J. & Williams, W. (2008) Maintaining group memberships: Social identity continuity predicts well‐being after stroke. Neuropsychological Rehabilitation, 18, 671–691. 10.1080/09602010701643449 18924001

[asap12275-bib-0051] Haslam, C. , Jetten, J. , Cruwys, T. , Dingle, G. , Haslam, S.A. , Jetten, J. , et al. (2018) The new psychology of health: Unlocking the social cure. Routledge. 10.4324/9781315648569

[asap12275-bib-0052] Hatzidimitriadou, E. (2002) Political ideology, helping mechanisms and empowerment of mental health self‐help/mutual aid groups. Journal of Community & Applied Social Psychology, 12(4), 271–285. 10.1002/casp.681

[asap12275-bib-0053] Hopkins, N. , Reicher, S. , Stevenson, C. , Pandey, K. , Shankar, S. & Tewari, S. (2019) Social relations in crowds: Recognition, validation and solidarity. European Journal of Social Psychology, 49(6), 1283–1297. 10.1002/ejsp.2586

[asap12275-bib-0054] Iyer, A. , Jetten, J. , Tsivrikos, D. , Postmes, T. & Haslam, S. (2009) The more (and the more compatible) the merrier: Multiple group memberships and identity compatibility as predictors of adjustment after life transitions. The British Journal of Social Psychology, 48, 707–733. 10.1348/014466608X397628 19200408

[asap12275-bib-0055] Independent SAGE (2020, Oct 30). Statement on the management of NHS test and trace. Available from: https://www.independentsage.org/statement‐on‐the‐management‐of‐nhs‐test‐and‐trace/

[asap12275-bib-0056] Jetten, J. , Haslam, C. , Haslam, S. , Dingle, G. & Jones, J. (2014) How groups affect our health and well‐being: The path from theory to policy. Social Issues and Policy Review, 8, 128. 10.1111/sipr.12003

[asap12275-bib-0057] Jetten, J. , Haslam, S. , Cruwys, T. , Greenaway, K. , Haslam, C. & Steffens, N.K. (2017) Advancing the social identity approach to health and well‐being: Progressing the social cure research agenda. European Journal of Social Psychology, 47, 789–802. 10.1002/ejsp.2333

[asap12275-bib-0058] Joffe, H. (2011) Thematic analysis. In: Harper, D. & Thompson, A. R. (Eds.), Qualitative methods in mental health and psychotherapy: A guide for students and practitioners. Chichester: John Wiley & Sons, Ltd., pp. 209–223. 10.1002/9781119973249.ch15

[asap12275-bib-0059] Kaniasty, K. & Norris, F. (1999) The experience of disaster: Individuals and communities sharing trauma. In: Gist, R. & Lubin, B. (Eds.), Response to disaster: Psychosocial, community and ecological approaches. Philadelphia, PA: Brunner/Mazel, pp. 25–61.

[asap12275-bib-0060] Kavada, A. (2020, June 12) Creating a hyperlocal infrastructure of care: COVID‐19 mutual aid groups. *Opendemoc*racy. Available from: https://www.opendemocracy.net/en/openmovements/creating‐hyperlocal‐infrastructure‐care‐covid‐19‐mutual‐aid‐groups/

[asap12275-bib-0061] Keeton, C.P. , Perry‐Jenkins, M. & Sayer, A.G. (2008) Sense of control predicts depressive and anxious symptoms across the transition to parenthood. Journal of Family Psychology, 22(2), 212–221. 10.1037/0893-3200.22.2.212 18410208 PMC2834184

[asap12275-bib-0062] Klandermans, P.G. (2014) Identity politics and politicized identities: Identity processes and the dynamics of protest. Political Psychology, 35(1), 1–22.

[asap12275-bib-0063] Klar, M. & Kasser, T. (2009) Some benefits of being an activist: Measuring activism and its role in psychological well‐being. Political Psychology, 30(5), 755–777. 10.1111/j.1467-9221.2009.00724.x

[asap12275-bib-0064] Landis, J.R. & Koch, G.G. (1977) The measurement of observer agreement for categorical data. Biometrics, 33(1), 159–174. 843571

[asap12275-bib-0065] Maslow, A.H. (1970) Motivation and personality (2nd ed). New York: Harper & Row.

[asap12275-bib-0066] McGregor, C. (2020) Coronavirus, community and solidarity. Concept, 11, 1–5.

[asap12275-bib-0067] Monbiot, G. (2020, March 31). The horror films got it wrong. This virus has turned us into caring neighbours. *The Guardian*. Available from: https://www.theguardian.com/commentisfree/2020/mar/31/virus‐neighbours‐covid‐19

[asap12275-bib-0068] Monforte, P. (2019) From compassion to critical resilience: Volunteering in the context of austerity. The Sociological Review, 68(1), 110–126. 10.1177/0038026119858220

[asap12275-bib-0069] Nicolas, B. (2017) Virtuous and vicious anger. Journal of Ethics and Social Philosophy, 11(3), 1–28. 10.26556/jesp.v11i3.112

[asap12275-bib-0070] Norris, F.H. , Stevens, S.P. , Pfefferbaum, B. , Wyche, K.F. & Pfefferbaum, R.L. (2008) Community resilience as a metaphor, theory, set of capacities, and strategy for disaster readiness. American Journal of Community Psychology, 41(1–2), 127–150. 18157631 10.1007/s10464-007-9156-6

[asap12275-bib-0071] Nosek, B.A. , Simonsohn, U. , Moore, D.A. , Nelson, L.D. , Simmons, J.P. , Sallans, A. , et al. (2017, June 12) Standard reviewer statement for disclosure of sample, conditions, measures, and exclusions. Open Science Framework. Available from osf.io/hadz3.

[asap12275-bib-0072] Ntontis, E. , Drury, J. , Amlôt, R. , Rubin, G.R. & Williams, R. (2020) Endurance or decline of emergent groups following a flood disaster: Implications for community resilience. International Journal of Disaster Risk Reduction, 45. 10.1016/j.ijdrr.2020.101493

[asap12275-bib-0073] O'Dwyer, E. (2020a, June 23) COVID‐19 mutual aid groups have the potential to increase intergroup solidarity—But can they actually do so? British Politics and Policy at LSE. Available from: https://blogs.lse.ac.uk/politicsandpolicy/covid19‐mutual‐aid‐solidarity/

[asap12275-bib-0074] O'Dwyer, E. (2020b) Solidarity not charity? A political psychological perspective on UK COVID‐19 Mutual Aid Groups (Conference Presentation).

[asap12275-bib-0075] Office for National Statistics (2020, April 3) Coronavirus and the social impacts on Great Britain: 3 April 2020. Office for National Statistics. Available from: https://www.ons.gov.uk/peoplepopulationandcommunity/healthandsocialcare/healthandwellbeing/bulletins/coronavirusandthesocialimpactsongreatbritain/23april2020

[asap12275-bib-0076] Parveen, N. & McIntyre, N. (2020, May 29) Britons think UK will be more united after coronavirus recovery. The Guardian. Available from: https://www.theguardian.com/world/2020/may/29/britons‐think‐uk‐will‐be‐more‐united‐after‐coronavirus‐recovery

[asap12275-bib-0077] Patton, M.Q. (2002) Two decades of developments in qualitative inquiry: A personal, experiential perspective. Qualitative Social Work, 1(3), 261–283. 10.1177/1473325002001003636

[asap12275-bib-0078] Plough, A. , Fielding, J.E. , Chandra, A. , Williams, M. , Eisenman, D. , Wells, K.B. , et al. (2013) Building community disaster resilience: Perspectives from a large urban county department of public health. American Journal of Public Health, 103(7), 1190–1197. 10.2105/AJPH.2013.301268 23678937 PMC3682619

[asap12275-bib-0079] Public Health England . (2020a) Beyond the data: Understanding the impact of COVID‐19 on BAME communities.

[asap12275-bib-0080] Public Health England . (2020b) Disparities in the risk and outcomes of COVID‐19.

[asap12275-bib-0081] Roberts, R.C. (1989) Aristotle on virtues and emotions. Philosophical Studies: An International Journal for Philosophy in the Analytic Tradition, 56(3), 293–306.

[asap12275-bib-0082] Rubin, G.J. , Smith, L.E. , Melendez‐Torres, G.J. & Yardley, L. (2020) Improving adherence to ‘test, trace and isolate’. Journal of the Royal Society of Medicine, 113(9), 335–338. 32910870 10.1177/0141076820956824 PMC7488807

[asap12275-bib-0083] Seebohm, P. , Chaudhary, S. , Boyce, M. , Elkan, R. , Avis, M. & Munn‐Giddings, C. (2013) The contribution of self‐help/mutual aid groups to mental well‐being. Health & Social Care in the Community, 21(4), 391–401. 10.1111/hsc.12021 23445336

[asap12275-bib-0084] Shevlin, M. , Butter, S. , McBride, O. , Murphy, J. , Gibson Miller, J. , Hartman, T.K. , et al. ((2021) February 23) Refuting the myth of a ‘tsunami’ of mental ill‐health in populations affected by COVID‐19: Evidence that response to the pandemic is heterogenous, not homogeneous. 10.31234/osf.io/ujwsm PMC8111207 33875044

[asap12275-bib-0085] Simon, B. & Klandermans, B. (2001) Politicized Collective Identity. American Psychologist, 13. 11330229

[asap12275-bib-0086] Smith, J.A. & Eatough, V. (2012) Interpreting phenomenological analysis. In: Breakwell , G. M. , Smith , J. A. & Wright , D. B. (Eds.), Research methods in psychology (4th ed.). Thousand Oaks, CA: Sage, pp. 439–459.

[asap12275-bib-0087] Smith, L.E. , Potts, H.W.W. , Amlot, R. , Fear, N.T. , Michie, S. & Rubin, J. (2020a) Adherence to the test, trace and isolate system: Results from a time series of 21 nationally representative surveys in the UK (the COVID‐19 Rapid Survey of Adherence to Interventions and Responses [CORSAIR] study). MedRxiv, 2020.09.15.20191957. 10.1101/2020.09.15.20191957

[asap12275-bib-0088] Smith, L.E. , Amlot, R. , Lambert, H. , Oliver, I. , Robin, C. , Yardley, L. , et al. (2020b) Factors associated with adherence to self‐isolation and lockdown measures in the UK: A cross‐sectional survey. Public Health, 187, 41–52. Advance online publication. 10.1016/j.puhe.2020.07.024 32898760 PMC7474581

[asap12275-bib-0089] Smith, L. , Thomas, E.F. & McGarty, C. (2015) We must be the change we want to see in the world”: Integrating norms and identities through social interaction. Political Psychology, 36(5), 543–557. 10.1111/pops.12180

[asap12275-bib-0090] SPI‐B . (2020a, Sept 16) Impact of financial and other targeted support on rates of self‐isolation or quarantine. Available from: https://www.gov.uk/government/publications/spi‐b‐impact‐of‐financial‐and‐other‐targeted‐support‐on‐rates‐of‐self‐isolation‐or‐quarantine‐16‐september‐2020

[asap12275-bib-0091] SPI‐B . (2020b, Nov 6) The role of Community Champions networks to increase engagement in the context of COVID‐19: Evidence and best practice. Available from: https://www.gov.uk/government/publications/spi‐b‐impact‐of‐financial‐and‐other‐targeted‐support‐on‐rates‐of‐self‐isolation‐or‐quarantine‐16‐september‐2020

[asap12275-bib-0092] Solnit, R. (2009) A paradise built in hell: The extraordinary communities that arise in disasters. Viking Adult.

[asap12275-bib-0093] Solnit, R. (May 14, (2020) The way we get through this is together’: Mutual aid under coronavirus. The Guardian, Available from: https://www.theguardian.com/world/2020/may/14/mutual‐aid‐coronavirus‐pandemic‐rebecca‐solnit

[asap12275-bib-0094] Spade, D. (2019) Mutual aid chart—Dean spade. Available from https://www.deanspade.net/2019/12/04/mutual‐aid‐chart/

[asap12275-bib-0095] Spade, D. (2020) Solidarity Not Charity: Mutual aid for mobilization and survival. Social Text, 38(1 (142)), 131–151. 10.1215/01642472-7971139

[asap12275-bib-0096] Stevenson, C. , Wakefield, J.R.H. , Felsner, I. , Drury, J. & Costa, S. (2021) Collectively coping with coronavirus: Local community identification predicts giving support and lockdown adherence during the COVID‐19 pandemic. British Journal of Social Psychology, 10.1111/bjso.12457 PMC8236966 33969899

[asap12275-bib-0097] Tajfel, H. & Turner, J. (1979) An integrative theory of intergroup conflict. In: Austin, W.G. & Worchel, S. (Eds.) The social psychology of intergroup relations. Monterey, CA: Brooks/Cole, pp. 33–48.

[asap12275-bib-0098] Tekin, S. & Drury, J. (2021) Silent walk as a street mobilization: Campaigning following the grenfell tower fire. Journal of Community & Applied Social Psychology, 10.1002/casp.2521

[asap12275-bib-0099] Theurer, K. & Wister, A. (2010) Altruistic behaviour and social capital as predictors of well‐being among older Canadians. Ageing & Society, 30(1), 157–181. 10.1017/S0144686X09008848

[asap12275-bib-0100] Tiratelli, L. & Kaye, S. (2020) Communities vs coronavirus: The rise of mutual aid. New Local Government Network.

[asap12275-bib-0101] Turner, J.C. , Hogg, M.A. , Oakes, P.J. , Reicher, S.D. & Wetherell, M.S. (1987) Rediscovering the social group: A self‐categorization theory. Oxford, UK: Blackwell.

[asap12275-bib-0102] United Nations . (2020) Policy brief: COVID‐19 and the need for action on mental health. Available from: https://unsdg.un.org/resources/policy‐brief‐covid‐19‐and‐need‐action‐mental‐health

[asap12275-bib-0103] Vestergren, S. , Drury, J. & Chiriac, E. (2016) The biographical consequences of protest and activism: A systematic review and a new typology. Social Movement Studies, 16(2). 10.1080/14742837.2016.1252665

[asap12275-bib-0104] Vestergren, S. , Drury, J. & Chiriac, E.H. (2019) How participation in collective action changes relationships, behaviours, and beliefs: An interview study of the role of inter‐ and intragroup processes. Journal of Social and Political Psychology, 7(1), 76–99. 10.5964/jspp.v7i1.903

[asap12275-bib-0105] Vignoles, V.L. , Jaser, Z. , Taylor, Z. & Ntontis, E. (2021) Harnessing shared identities to mobilise resilient responses to the COVID‐19 pandemic. Political Psychology, 10.1111/pops.12726. PMC8013210 33821062

[asap12275-bib-0106] Wakefield, J.R.H. , Bowe, M. , Kellezi, B. , McNamara, N. & Stevenson, C. (2019) When groups help and when groups harm: Origins, developments, and future directions of the “Social Cure” perspective of group dynamics. Social and Personality Psychology Compass, 13(3). 10.1111/spc3.12440

[asap12275-bib-0107] Waterman, A.S. (1993) Two conceptions of happiness: Contrasts of personal expressiveness (eudaimonia) and hedonic enjoyment. Journal of Personality and Social Psychology, 64(4), 678–691. 10.1037/0022-3514.64.4.678

[asap12275-bib-0108] Webster, R.K. , Brooks, S.K. , Smith, L.E. , Woodland, L. , Wessely, S. & Rubin, G.J. (2020) How to improve adherence with quarantine: Rapid review of the evidence. Public Health, 182, 163–169. 10.1016/j.puhe.2020.03.007 32334182 PMC7194967

[asap12275-bib-0109] Wein, T. (2020) How mutual aid might change Britain. Dignity Project. Available from: https://dignityproject.net/our‐research/mutual‐aid‐in‐the‐uk/

[asap12275-bib-0110] Xu, X. & Banks, J. (2020) The mental health effects of the first two months of lockdown and social distancing during the Covid‐19 pandemic in the UK. The IFS. 10.1920/wp.ifs.2020.1620

[asap12275-bib-0111] Zhou, X. & Yao, B. (2020) Social support and acute stress symptoms (ASSs) during the COVID‐19 outbreak: Deciphering the roles of psychological needs and sense of control [Soporte social y sintomas de estres agudo (SEAs) durante el brote del COVID‐19: decifrando los roles de las necesidades psicológicas y la sensación de control. European Journal of Psychotraumatology, 11(1), 1779494. 10.1080/20008198.2020.1779494 33062202 PMC7534382

